# DNA Damage as a Mechanistic Link between Air Pollution and Obesity?

**DOI:** 10.3390/medicines10010004

**Published:** 2022-12-29

**Authors:** Abdelaziz Ghanemi, Mayumi Yoshioka, Jonny St-Amand

**Affiliations:** 1 Department of Molecular Medicine, Faculty of Medicine, Laval University, Québec, QC G1V 0A6, Canada; 2 Functional Genomics Laboratory, Endocrinology and Nephrology Axis, CHU de Québec-Université Laval Research Center, Québec, QC G1V 4G2, Canada

**Keywords:** air pollution, obesity, DNA damage

## Abstract

It has been shown that the risk of developing obesity, a serious modern health problem, increases with air pollution. However, the molecular links are yet to be fully elucidated. Herein, we propose a hypothesis via which air pollution-induced DNA damage would be the mechanistic link between air pollution and the enhanced risk of obesity and overweight. Indeed, whereas air pollution leads to DNA damage, DNA damage results in inflammation, oxidative stress and metabolic impairments that could be behind energy balance changes contributing to obesity. Such thoughts, worth exploring, seems an important starting point to better understand the impact of air pollution on obesity development independently from the two main energy balance pillars that are diet and physical activity. This could possibly lead to new applications both for therapies as well as for policies and regulations.

Obesity represents one of the most challenging health problems for modern societies [1,2]. Its basic definition is an accumulation of extra energy intake resulting from a high food intake and/or insufficient energy expenditure [3]. The pathogenesis and underlying mechanisms are complex and involve neuroendocrine component [4], genetics [5], broken energy balance [3], biochemical environment variations [6], among others. Obesity has even been compared to cancer in terms of tissue distribution and development [7], and it has also been considered as a disease [8,9]. The most serious issues about obesity are those related to its health impacts. It represents a risk factor for a variety of diseases and health problems [10,11,12,13,14,15,16,17]. Obesity also impairs regeneration [18,19]. Furthermore, in the context of coronavirus disease-2019 (COVID-19) crisis, a spotlight has been put on obesity by health experts since obesity is a risk factor for sever forms of COVID-19 and the measures taken by health authorities to limit COVID-19 spread could have worsened obesity pandemic [20,21,22,23].

Obesity research mainly focuses on two well-characterized key factors in obesity development, which are, respectively, energy intake and energy expenditure. Both diet and physical activity are at the center of obesity management [24]. Indeed, these two are the factors the individual can control to a big extend by following a healthy lifestyle including a balanced diet [25,26] and sufficient physical activity [27]. Other non-caloric factors that also impact obesity development such as sleeping cycle and psychological status can be managed as well.

However, there are factors that individuals cannot control. These factors can be internal or external. While the most known internal factor is genetics, a good example of external factors impacting obesity development is the air pollution. Indeed, among the interesting external factors that has been shown as involved in obesity and that are also outside the control of individuals is the air pollution. Links between pollution and obesity have been studied and pointed air pollution as a cause of obesity [28] or as increasing the risk of both overweight and obesity [29]. Air pollution includes components such as nitrogen oxides, ultrafine particles, ozone, carbon monoxide and polyaromatic hydrocarbons [28,29,30,31].

Herein, this piece of writing aims to provide a hypothesis to explain the mechanistic links between pollution and obesity via the DNA damage. Strong evidence from the literature point that those pollutants induce DNA damage [31,32,33,34,35,36,37] and air pollution modulates both DNA methylation and epigenetic mark [30]. For instance, in vivo studies showed that nitrogen dioxide induces dose- and time-dependent DNA single-strand breaks [38] and exposing animals to air pollution increases oxidized guanines in the lung [39]. It is worth noting that, within this context, DNA damage-related measures have even been suggested as biomarkers for air pollution impacts on health [33,35,40].

Knowing that obesity includes among its molecular markers epigenetic changes [41], we come with the following theory to explain links between air pollutants and obesity development. The DNA damage seen during obesity would not only be the consequence of obesity, via oxidative stress and inflammation, [42] but also either contribute to obesity development as previously described [43] or at least develop in parallel with obesity and influence obesity-related patterns such as inflammation and diseases risk development [30]. Based on that, DNA damage could be the missing link in the mechanistic chain of events. Indeed, air pollutant would lead to DNA damage which would contribute to develop obesity or increase its risk (Figure 1). Such hypothesis is supported by the fact that air pollution associated with DNA damage is also associated with metabolic alterations including purine, pyrimidine and glycolysis/gluconeogenesis perturbed metabolism [37] as well as disturbances in the biosynthesis of unsaturated fatty acids and metabolism of glycerophospholipid, propanoate, sphingolipid, beta-alanine, glutathione and pyruvate especially when the air pollution is combined with temperature [44,45].

Such biochemical consequences of exposure to air pollutant could indicate a metabolism towards a disturbed energy balance potentially contributing to obesity development. This is not contradictory with the direct relation between obesity and DNA damage as shown in mice in which weigh loss was accompanied by decrease in DNA damage [46]. Therefore, our hypothesis does not say that obesity does not lead to DNA damage, but it says that DNA damage might also occur before and during obesity development (as a result of exposure to air pollutants), contributes to obesity development and increases the health risks associated with obesity in terms of cancer and metabolic disorders among other health problems [47,48]. In addition, air pollutions also cause both inflammation and oxidative stress [32], which would further exacerbate the inflammation-related and oxidative stress-related consequences of obesity.

Although an individual living in a polluted area cannot control the impact of air pollution on obesity development, such theory—if further explored—would lead to a better understanding of how DNA damage leads to develop obesity. Most importantly, this theory is towards developing more concrete measures of how deeply air pollutants impact obesity development and which pollutants have the deepest impacts. Within this context, and to complete the data linking air pollution to obesity within a population, in vitro studies and animal experiments can be conducted. It would consist of exposing animals as well as in vitro samples (cells cultures, tissues, etc.) to the different air pollutants with different pollutants combinations and concentrations. After such exposures, measures related to DNA damage will be completed to evaluate the impacts of the studied air pollutants in the context of obesity development. A wider biological study can be performed to also explore other biological entities as well as the proteins, pathways and functions controlled or interacting with genes impacted by the DNA damage in order to have a more panoramic view of the mechanistic pathways at the cellular and molecular levels, as illustrated by the air pollution-induced lipid oxidative damage [35]. In addition, exposing the animal models of obesity [49,50] to air pollutants could optimize these models toward a better mimicking of obesity development.

Beyond developing our understanding of air pollution-obesity links, such studies will provide precise data for decisions makers and politicians. It is of a particular importance when it comes—for instance—to decide where factories should be built. Whether industries should be hold responsible for the health impacts of the air pollutants they produce depending on the concentrations they produce (set legal emission limits) is another important application.

## Figures and Tables

**Figure 1:**
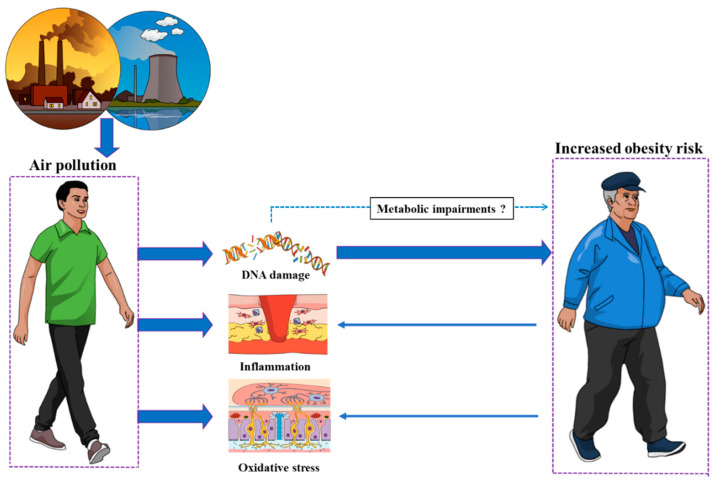
Air pollution increases obesity development risk hypothetically via the DNA damage that pollution induces. DNA damage-related metabolic impairments might also be involved.

## Data Availability

Not applicable.
